# Grapevine LysM-containing receptor-like kinase *VvLYK6* enhances resistance to white rot in tomato

**DOI:** 10.3389/fpls.2026.1869419

**Published:** 2026-06-09

**Authors:** Qibao Liu, Weixu Wang, Zichao Chen, Nanyang Li, Tinggang Li

**Affiliations:** 1 Shandong Academy of Grape, Shandong Academy of Agricultural Sciences, Jinan, China; 2 School of Landscape and Ecological Engineering, Hebei University of Engineering, Handan, China

**Keywords:** grapevine, LysM-containing receptor-like kinases, resistance mechanism, SA signaling pathway, white rot

## Abstract

Grapevine (*Vitis vinifera*) is a fruit that is widely cultivated worldwide, but white rot, caused by *Coniella vitis*, significantly impacts its yield and quality. The lack of resistance genes poses a major challenge to breeding grapevine varieties resistant to white rot. The LysM-containing receptor-like kinase (LYK) family genes are known to mediate immune responses to pathogens by recognizing pathogen-associated molecular patterns. To explore the resistance genes underlying white rot resistance, whole-transcriptome sequencing was carried out on grapevine cv. Guifeimeigui (GF, resistant phenotype) and cv. Red Globe (RG, susceptible phenotype) after challenge with *C. vitis*. Functional characterization and mechanistic analysis were conducted through virus-induced gene silencing (VIGS) in grapevine, stable overexpression in grapevine calli, and heterologous expression in tomato (*Solanum lycopersicum*). Differentially expressed genes (DEGs) enriched in the “Plant-pathogen interaction” pathway were identified, followed by expression clustering analysis to pinpoint 14 candidate genes. Silencing of *VvLYK6* in GF resulted in a significant reduction in resistance to white rot, while its overexpression in grapevine calli inhibited the growth of *C. vitis*. *VvLYK6* is localized to the cell membrane. SlLYK1 was involved in the resistance to *C. vitis* in tomato that was mediated by *VvLYK6*. The overexpression of *VvLYK6* in tomato also activated the salicylic acid signaling pathway and upregulated related pathogenesis-related genes, moreover induced a burst of reactive oxygen species, thereby increasing resistance to white rot. This study identifies *VvLYK6* as a critical regulator of white rot resistance, and reveals its immune mechanism, and highlights its potential as a target for breeding and genetic improvement of durable resistance in grapevine.

## 1 Introduction

Receptor-like kinases (RLKs) are crucial membrane proteins in plants that recognize pathogen-associated molecular patterns (PAMPs) and initiate immune responses (Yuan et al., 2021). The RLK family is divided into several subfamilies based on the features of their extracellular domains and the presence of intracellular kinase domains (Boutrot and Zipfel, 2017). The LysM-containing receptor-like kinases (LYKs) subfamily, which includes an extracellular LysM domain, a transmembrane region, and an intracellular kinase domain, is particularly important (Brulé et al., 2019). The LysM domain, with its conserved CxC structure, forms disulfide bonds that link LysM molecules. This enables the recognition of PAMPs that contain structures of N-acetylglucosamine, thereby facilitating plant-pathogen interactions (Gust et al., 2012). The transmembrane region plays a critical role in the localization of proteins and the intracellular transmission of immune signals mediated by oligomerization (Morillo and Tax, 2006). The intracellular kinase domain contains multiple catalytic sites that initiate phosphorylation and dephosphorylation processes, promote protein-substrate binding and activate immune signaling pathways, thereby mediating plant resistance responses (Antolín-Llovera et al., 2012).

To date, LYK family genes have been reported to be involved in immune responses that are induced by chitin (Ai et al., 2023; Chen et al., 2020a; Ren et al., 2022; Su et al., 2026). *Arabidopsis thaliana* contains five AtLYK family genes, with AtLYK1/CERK1 serving as the central component of the chitin receptor complex by interacting with AtLYK5 (Cao et al., 2014; Erwig et al., 2017). AtLYK4 also binds chitin but lacks kinase activity, and its interaction with CERK1 depends on AtLYK5 (Wan et al., 2012). StCERK1 is also identified as a functional kinase essential for chitin signaling and contributed to the resistance of potato (*Solanum tuberosum*) to the *Phytophthora infestans*, *Alternaria solani*, and *Ralstonia solanacearum* (Cao et al., 2025). In cotton (*Gossypium hirsutum*), GhLYK5, the closest ortholog of AtLYK5, physically interacts with nitrilase GhNIT4B, which activates asparagine and ROS accumulation and further enhances cotton *Verticillium wilt* resistance (Li et al., 2026). In wheat (*Triticum aestivum*), TaLYK5 plays a positive regulatory role in the wheat-*Puccinia striiformis* interaction, and its effect can be suppressed by miRNA tae-miR1714 (Xiao et al., 2025). AtLYK2 enhances resistance to *Botrytis cinerea* and *Pseudomonas syringae* by positively regulating defense gene expression, while AtLYK3 negatively regulates these genes, thereby mediating resistance to *B. cinerea* and *Pectobacterium carotovorum* (Giovannoni et al., 2021; Paparella et al., 2014). Homologs of the *A. thaliana* chitin receptor components *CERK1*, *AtLYK4* and *AtLYK5* have been identified in grapevine, encompassing the *VvLYK1*, *VvLYK4*, and *VvLYK5*. Functional complementation assays demonstrate that *VvLYK1–1* rescues *Atcerk1* mutants, whereas *VvLYK4–2* and *VvLYK5–1* complement *Atlyk4/5* double mutants. Heterologous expression of these genes restores chitin- and/or chitosan-triggered MAPK activation, defense gene induction, and callose deposition, ultimately conferring resistance to Erysiphe necator (Brulé et al., 2019; Roudaire et al., 2023, 2026). Collectively, these findings establish *VvLYK1-1*, *VvLYK4-2*, and *VvLYK5–1* as evolutionarily conserved core receptors that transduce cell wall-derived elicitor perception into canonical immune signaling to orchestrate pathogen resistance. However, the role of VvLYK family genes in resistance to white rot disease in grapevine has not yet been explored.

Salicylic acid (SA) is an important plant hormone that not only regulates various growth and developmental processes but also plays a vital role in defending against pathogens (Vlot et al., 2009). In mutants deficient in SA biosynthesis, the responses of both PAMP-triggered immunity (PTI) and effector-triggered immunity (ETI) to pathogens are severely compromised (Jones and Dangl, 2006). The regulation of plant growth, development, and environmental stress responses by SA is achieved by altering the concentrations of SA and the expression of downstream genes associated with this hormone (Tsuda et al., 2009). NPR1, a master regulator of SA signaling, undergoes conformational changes upon SA perception that promote its nuclear accumulation (Wu et al., 2012). Within the nucleus, NPR1 interacts with the transcription factor TGA1 to form a nucleoprotein complex that binds to the promoter regions of pathogenesis-related genes, including *PR1* and *PR5*, thereby activating their transcription (Després et al., 2000; Fan and Dong, 2002). The coordinated expression of these genes provides a robust and widely accepted indicator of immune activation through the SA signaling pathway (Fan and Dong, 2002). Upon infection by pathogens, the biosynthesis and signal transduction of SA are enhanced, which induces the expression of defense-related genes and improves plant resistance (Seyfferth and Tsuda, 2014). The isochorismate synthase (ICS) pathway and phenylalanine ammonia-lyase (PAL) pathway are the two main routes of SA biosynthesis in plants, with immune-related SA primarily produced via the ICS pathway (Garcion et al., 2008; Huang et al., 2010; Strawn et al., 2007). In grapevine cv. Thompson Seedless, the accumulation of H_2_O_2_ and callose is induced by the overexpression of *VpCDPK9*/*VpCDPK13* to activate the SA signaling pathway, which mediates resistance to powdery mildew (Hu et al., 2021). VqWRKY31 induces a burst of reactive oxygen species (ROS) and the expression of pathogenesis-related (PR) genes via the SA signaling pathway, thereby mediating resistance to powdery mildew (Yin et al., 2022). VaRPP13 mediates resistance to downy mildew via the SA signaling pathway (Chen et al., 2022). VvPR1 recruits transcription factors to the promoter region, which promotes the expression of *VvPR1* after SA treatment, thus, mediating resistance to white rot (Rahman et al., 2022). However, the signaling pathways activated by the VvLYK family genes in the resistance of grapevine to pathogens remain unclear.

Grapevine is a globally cultivated species of fruit with a long history of cultivation (Li et al., 2023b). White rot, caused by *Coniella vitis*, is one of the most destructive diseases affecting global grapevine production. The pathogen primarily spreads via rain-splash and wind, enters the host through natural wounds, insect damage, or direct epidermal penetration, and is capable of infecting all aerial tissues of the grapevine, with particularly severe impacts on clusters, shoots, and leaves (Zhang et al., 2023b). Current studies on grapevine resistance to white rot have yielded some progress. Studies have shown that the *C. vitis* effector CdE1 can bind to VdCRK10 in spine grapevine (*V. davidii*), which reduces its resistance to white rot by inhibiting the accumulation of VdCRK10 (Liu et al., 2024). VvWRKY5 can inhibit the activity of the VvJAZ2 promoter, while promoting the activity of the VvMYC2 promoter. This reaction positively regulates the resistance of grapevine to white rot via the JAZ-MYC module in the jasmonic acid (JA) signaling pathway (Zhang et al., 2023b). A quantitative trait locus (QTL) related to white rot resistance was identified on linkage group LG14 through the interspecific hybridization of resistant and susceptible grapevine varieties, explaining 12.93%-13.43% of the phenotypic variation. This led to the development of a single nucleotide polymorphism (SNP) marker that co-segregated with resistance to white rot (Su et al., 2021). The whole-genome sequencing of the ‘Manicure Finger’ and ‘0940’ grapevine parents, along with 101 F1 progenies, identified a stable QTL for white rot resistance on chromosome 3, which explained 17.9% of the phenotypic variation (Li et al., 2023a). Additionally, genes, such as *VvNPR1, VvTGA4, VvPti6*, and *VvMYC2*, in grapevine have been reported to potentially mediate resistance to white rot disease (Su et al., 2019).

In this study, the transcriptome sequencing of grapevine varieties with contrasting responses to inoculation with *C. vitis* revealed a significant enrichment of differentially expressed genes (DEGs) in the “Plant-pathogen interaction” pathway. A clustering analysis of DEGs identified candidate genes, and a virus-induced gene silencing (VIGS) analysis determined that the *VvLYK6* gene mediates resistance to white rot. VvLYK6 is localized to the cell membrane, and its overexpression in grapevine calli significantly inhibited the growth of *C. vitis*. The overexpression of *VvLYK6* in tomato (*Solanum lycopersicum*) also enhanced resistance to white rot, and *SlLYK1* played an indispensable role. Further analysis in tomato revealed that VvLYK6 induces the ROS burst and activates the SA signaling pathway and pathogenesis-related (PR) genes, mediating resistance to white rot.

## 2 Materials and methods

### 2.1 Sample preparation and transcriptome sequencing

Two-year-old grapevine seedlings of GF (resistant phenotype) and RG (susceptible phenotype) were grown in a greenhouse at the Shandong Academy of Grape, Jinan, Shandong, China (36°65′ N, 117°07′ E), under controlled conditions of a 16 h light/8 h dark photoperiod, 25 °C, and 95% relative humidity. The GP1 isolate of *C. vitis* was cultured in liquid sporulation medium for 9 days, and the conidia were then collected and diluted to a concentration of 1×10^2^ conidia/mL in deionized water. The grapevine leaves were inoculated with the spore suspension, and samples were collected at 6, 12, 24, 36, 48, and 72 h post-inoculation. Deionized water treated grapevine leaves served as the controls (Wan et al., 2021). Each experiment included three biological replicates, with six leaves sampled per replicate. The total RNA was extracted using the CTAB pBIOZOL reagent kit (Bioflux, Cell Microsystems, Durham, NC, USA) and its quality and quantity were assessed using a NanoDrop spectrophotometer (Thermo Fisher Scientific, Waltham, MA, USA) and an Agilent 2100 bioanalyzer (Agilent Technologies, Santa Clara, CA, USA). High-quality RNA samples were used for library construction and transcriptome sequencing. In the pathogenicity assays, detached leaves of grapevine and tomato were inoculated with mycelium plugs (5 mm diameter), and controls were inoculated with PDA plugs (5 mm diameter). All treated grapevine leaves were placed in a greenhouse maintained at 25°C and 95% relative humidity. The infection rate and lesion diameter were measured 3 days after inoculation.

### 2.2 Selection and analysis of the differentially expressed genes

The clean reads obtained after quality control were aligned to the reference genome PN40024 12X.v2 using HISAT2 software (**Supplementary Table 1**). The transcripts were quantified using feature counts, and they were normalized to obtain the fragments per kilobase of transcript per million mapped reads (FPKM) value for each gene. All the expressed genes were subjected to a principal component analysis (PCA) and visualized using the ggplot2 package in R software. The DEGs were analyzed using DESeq2 software with a threshold of |log_2_(fold changes)| ≥ 1 and adjusted p-value ≤ 0.05. The DEGs were clustered based on their patterns of gene expression using the K-means method, and Gene Ontology (GO) and Kyoto Encyclopedia of Genes and Genomes (KEGG) pathway enrichment analyses were conducted using the ClusterProfiler package in R. A clustering analysis of the levels of transcription of significantly enriched genes in the candidate pathways was performed. The RNA-seq data were deposited in the NCBI SRA database as accession numbers PRJNA995417 and PRJNA1001063.

### 2.3 Virus-induced gene silencing analysis

Candidate gene-specific fragments were amplified from the GF cDNA and integrated into the pTRV2 vector to construct pTRV2:CGs (**Supplementary Table 2**). The fusion vectors were introduced into *Agrobacterium tumefaciens* GV3101. The *A. tumefaciens* culture was shaken in LB media, centrifuged at 4 °C and 12, 000×g for 5 min, and the cells were resuspended in injection buffer to an OD_600_ = 1.2. The *A. tumefaciens* strains that harbored pTRV2:CGs and pTRV1 vectors were mixed in a ratio of 1:1, v/v, and the suspension was injected into grapevine or tomato leaves. The pTRV2:00 vector served as a control, and the pTRV2:*VvPDS* vector was used to evaluate the efficacy of VIGS. We selected 36 day-old GF plants planted in a greenhouse for seed germination and injected them with syringes. The plants were grown under standard greenhouse conditions for 14 or 28 days, and the bleached leaves of the *VvPDS* gene-silenced line were observed. The silencing efficiency of candidate genes was evaluated by quantitative reverse transcription PCR (RT-qPCR), with the pTRV:00 lines serving as the control (GF-CK).

### 2.4 Subcellular localization of VvLYK6

The coding sequence of *VvLYK6* was inserted into the pBGFP4 and pRTL2 vectors and fused with the *GFP* sequence to construct pBGFP4:*VvLYK6* and pRTL2:*VvLYK6*, respectively (**Supplementary Table 3**). A vector that only harbored the GFP sequence served as a control. Expression of the fusion constructs was driven by the 35S promoter and terminated by an Nos terminator. The pBGFP4:*VvLYK6* fusion vector was introduced into *A. tumefaciens* GV3101. After shaking incubation, the bacterial cells were collected by centrifugation and resuspended in injection buffer to an OD_600_ = 0.6 as described in Section 2.3. The resulting suspension was injected into tobacco (*Nicotiana benthamiana*) leaves to transiently express the fusion protein. Additionally, the pRTL2:VvLYK6 fusion vector was transiently expressed in onion (*Allium cepa*) epidermal cells using the biolistic PDS-1000/He particle delivery system (Bio-Rad, Hercules, CA, USA). The subcellular localization of the fusion protein was observed using laser scanning confocal microscopy (LSMT-PMT) with an excitation wavelength of 488 nm and an emission wavelength of 510 nm.

### 2.5 Genetic transformation of the grapevine calli and leaves

The coding sequence of the *VvLYK6* gene was inserted into the pCHF3 overexpression vector to construct pCHF3:*VvLYK6* (**Supplementary Table 3**). The fusion vector was introduced into *A. tumefaciens* GV3101. Grapevine cv. Thompson seedless calli were transformed using the *Agrobacterium*-mediated method as described by Shu et al (Shu et al., 2021), with selection on standard MS medium that contained 300 mg·L^−^¹ cefotaxime and 50 mg·L^−^¹ kanamycin. Plasmid constructs and the empty vector were introduced into *A. tumefaciens* strain GV3101 via electroporation. The transformants were cultured in LB liquid medium at 28 °C with shaking at 150 rpm for 12 h. Bacterial cells were collected by centrifugation at 9, 000 × g for 6 min and resuspended in infiltration buffer (10 mM MES, pH 5.6; 10 mM MgCl_2_; 200 µM acetosyringone), then adjusted to an OD_600_ of 0.8. Grapevine leaves were infiltrated by vacuum infiltration with the *Agrobacterium* suspension for 25 min. The infiltrated leaves were placed in culture dishes under humid conditions (Zhu et al., 2022). RT-qPCR was used to detect the level of expression of *VvLYK6* in the transformed calli and leaves.

### 2.6 Overexpression of VvLYK6 in tomato

The coding sequence of *VvLYK6* was inserted into the pBI121-FLAG transformation vector to construct pBI121:*VvLYK6* (**Supplementary Table 3**). The recombinant vector was introduced into *A. tumefaciens* EHA105, and tomato cv. Micro-Tom cotyledon explants were transformed using the *Agrobacterium*-mediated method (Li et al., 2021b). The transformed plants were grown on MS medium with kanamycin (50 mg/L), and the T1 generation seedlings were transplanted into pots for cultivation. All the potted tomato plants were grown under standard greenhouse conditions. Total RNA and protein were extracted from freshly harvested and liquid nitrogen-ground tomato leaves following Section 2.1 and Li et al. (2025). The *VvLYK6* overexpression lines were validated by RT-qPCR and Western blot using anti-FLAG antibodies (Li et al., 2025).

### 2.7 Measurement of the content of salicylic acid in the tomato leaves

The tomato leaves were sprayed with a conidial suspension of *C. vitis* strain GP1 (1×10^2^ conidia/mL), and samples were collected after 24 h post-inoculation. The samples were rapidly frozen in liquid nitrogen, ground thoroughly, and extracted using high-performance liquid chromatography (HPLC)-grade methanol. The extracts were dried using a vacuum centrifuge and dissolved in methanol. The content of SA was measured using HPLC (Waters, Milford, MA, USA), with a flow rate of 0.8 mL/min, a column temperature of 40 °C, and a detection wavelength of 230 nm. A standard curve was established, and the experiments were performed in triplicate.

### 2.8 Detection of reactive oxygen species burst in the tomato leaves

The tomato leaves were inoculated with *C. vitis* strain GP1 as described in Section 2.7, and samples were collected after 12 h post-inoculation. Hydrogen peroxide and superoxide anions in the tomato leaves were stained using 3, 3-diaminobenzidine (DAB) and nitroblue tetrazolium chloride (NBT), respectively. The H_2_O_2_ content and superoxide anion generation rate were measured using kits (Cat#BC3595 and Cat#BC1290, respectively) from Solarbio Life Sciences (Beijing, China).

### 2.9 Analysis of the patterns of gene expression

The expression pattern of *VvLYK6* was also analyzed in the GF leaves treated with a conidial suspension of *C. vitis* (1×10^2^ conidia/mL), as well as in leaves treated with solutions of chitin (0.1 g/L) and chitosan (0.1 g/L) provided by Elicityl (Crolles, France), with untreated GF leaves serving as the control. Samples were collected at 6, 12, 24, 36, 48, and 72 h post-inoculation. The tomato lines that overexpressed *VvLYK6* were used as the experimental groups, and tomato wild-type plants served as the control. After inoculation with the *C. vitis* GP1 strain for 24 h, samples were collected, and the expression levels of genes related to SA biosynthesis and PR genes were determined. RT-qPCR was performed using a QuantStudio 6 Flex real-time fluorescence quantitative PCR system (Thermo Fisher Scientific). The amplification conditions were as follows: initial denaturation at 95 °C for 10 min, denaturation at 95 °C for 15 s, annealing at 60 °C for 30 s, and extension at 72 °C for 20 s, for 40 cycles. V. vinifera *Actin-7* (XM_002282480) and *S. lycopersicum GAPDH* (U97257) were used as the internal reference genes, and gene expression levels were calculated using the 2^−ΔΔCT^ method (Li et al., 2023b; Yin et al., 2022). The primers used for RT-qPCR are listed in **Supplementary Table 4**.

## 3 Results

### 3.1 Transcriptome sequencing and analysis of the response of grapevine to white rot

**Figure 1:**
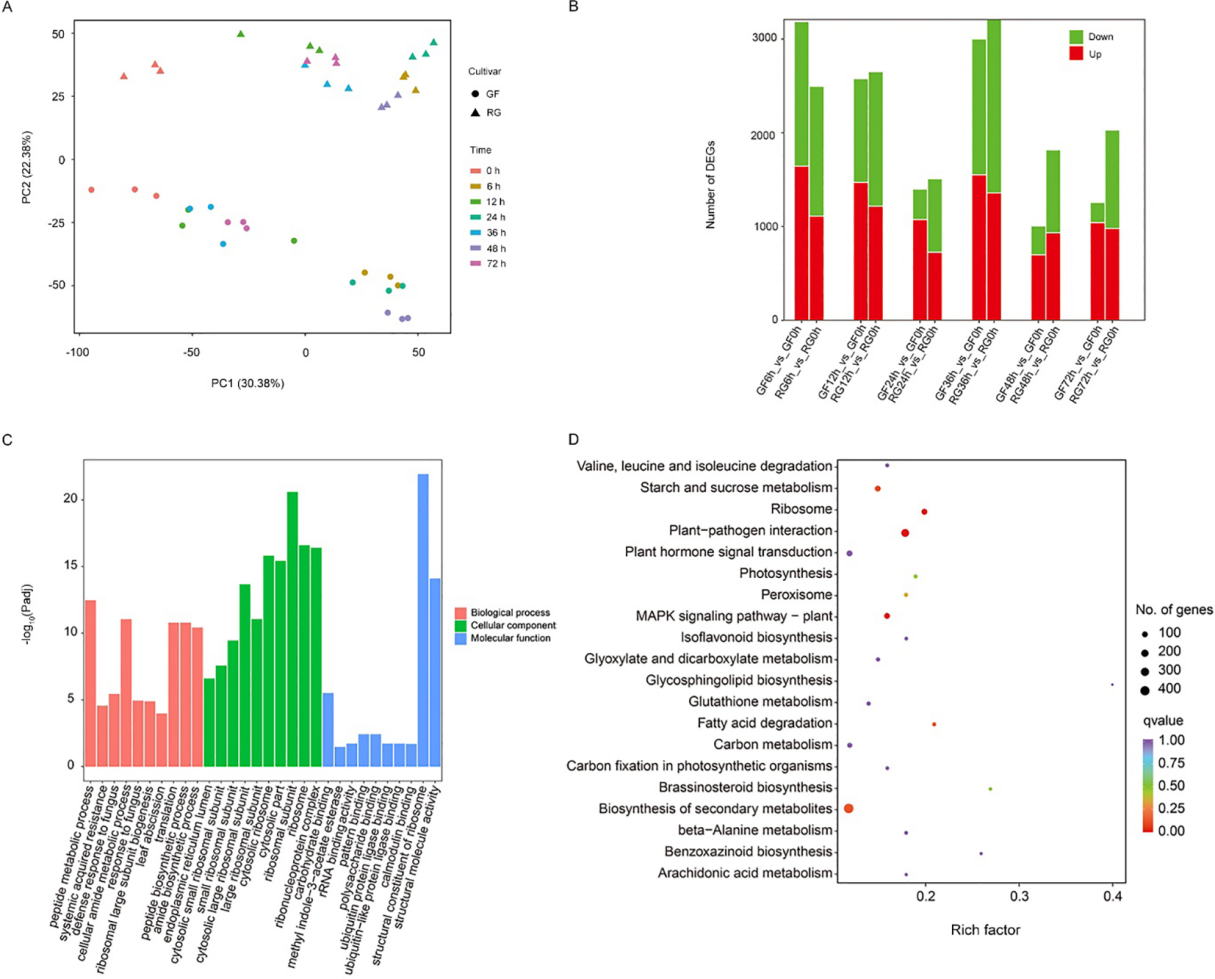
Transcriptome sequencing and analysis of the grapevine response to inoculation with *C. vitis*. **(A)** Principal component analysis (PCA) plot of all samples. Percentages represent the proportion of variance explained by each principal component. Each group consists of three samples, and samples in the same group are represented by the same color. **(B)** Bar chart of differentially expressed genes (DEGs). The green and red bars represent the number of down-regulated and up-regulated genes, respectively. **(C)** Gene ontology (GO) analysis of grapevine DEGs in response to *C. vitis*. Padj denotes the adjusted p-adjust. **(D)** Kyoto Encyclopedia of Genes and Genomes (KEGG) analysis of DEGs enriched in the term “response to fungus”. Black dots are used to mark the number of genes.

Three days after inoculation with *C. vitis*, the GF exhibited a lesion of only 1.01 cm in diameter, while the RG exhibited a lesion of 3.57 cm in diameter. These differences demonstrated the contrasting phenotypes of resistance and susceptibility to white rot, respectively (**Supplementary Figure 1**). The transcriptomes of GF and RG were sequenced at 6, 12, 24, 36, 48, and 72 h post-inoculation with *C. vitis*. The PCA results demonstrated high reproducibility among experimental samples collected at the same time point, with a marked separation observed between resistant and susceptible grapevine varieties. Additionally, within the same grapevine variety, samples collected at different time points post-inoculation displayed significant variation (**Figure 1A**; **Supplementary Tables 5**, **6**). The highest numbers of DEGs were at 6, 12, and 36 h post-inoculation with *C. vitis* (**Figure 1B**). The K-means clustering of all DEGs (6, 525) identified gene modules with distinct transcriptional patterns, among which cluster 4 and 5 displayed pronounced differences between GF and RG (**Supplementary Figure 2**). GO enrichment analysis of DEGs (2, 212) within these clusters revealed a significant enrichment of the term “response to fungus” (**Figure 1C**). Further KEGG pathway analysis of genes (136) within this term revealed a significant enrichment in the “plant-pathogen interaction” pathway (**Figure 1D**).

### 3.2 Virus-induced gene silencing analysis of the candidate genes

**Figure 2:**
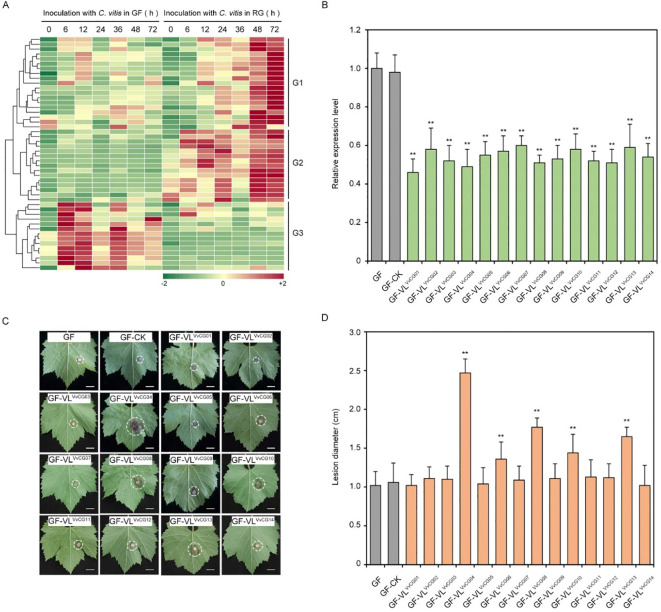
Expression patterns of candidate genes and the virus-induced gene silencing analysis. **(A)** Clustering analysis of differentially expressed genes enriched in the “plant-pathogen interaction” pathway. **(B)** Expression of *V. vinifera* candidate genes (VvCGs) in GF, the empty vector silenced line (GF-CK), and VvCGs-silenced (GF-VL^VvCGs^) lines. **(C)** Phenotypes of GF, GF-CK, and GF-VL^VvCGs^ lines after 72 h inoculation with *C. vitis*. **(D)** Statistical analysis of lesion diameter in GF, GF-CK, and GF-VL^VvCGs^ lines after 72 h inoculation with *C. vitis*. RT-qPCR was used to detect candidate gene expression levels, which were calculated using the 2^-ΔΔCT^ method with *V. vinifera Actin-7* as the internal reference gene. White dashed circles are used to mark the boundaries of diseased areas on grapevine leaves. Scale bar = 1 cm. Error bars represent the standard error of three biological replicates, and the double asterisks represents a significant difference at the P < 0.01 level by t-test.

The clustering analysis of the 48 significantly enriched genes in the “plant-pathogen interaction” pathway revealed that genes in the branch G3 were significantly upregulated in GF upon *C. vitis* inoculation, whereas their expression levels decreased in RG (**Figure 2A**; **Supplementary Table 7**). A total of 14 genes in branch G3 were designated as *V. vinifera* candidate genes 01-14 (VvCG01-14) based on their physical locations on the chromosomes. The VIGS analysis in GF showed that the silencing efficiency of the 14 candidate genes ranged from 40% to 56% (**Figure 2B**). The silencing of VvCG04 (GF-VL^VvCG04^) significantly compromised resistance to white rot, with lesion diameters that were 2.42- and 2.33-folds larger than those of the GF and GF-CK lines, respectively (**Figures 2C, D**). The gene VvCG04 was annotated as a member of the LysM-containing receptor-like kinase (LYK) family and named *VvLYK6* in this study. Phylogenetic analysis showed that most grapevine VvLYK genes clustered with direct orthologs from *A. thaliana*, *S. lycopersicum*, and *Oryza sativa* with strong bootstrap support, suggesting strong evolutionary constraint and conserved molecular functions (**Supplementary Figure 3**; **Supplementary Table 8**).

### 3.3 Subcellular localization analysis of VvLYK6

**Figure 3:**
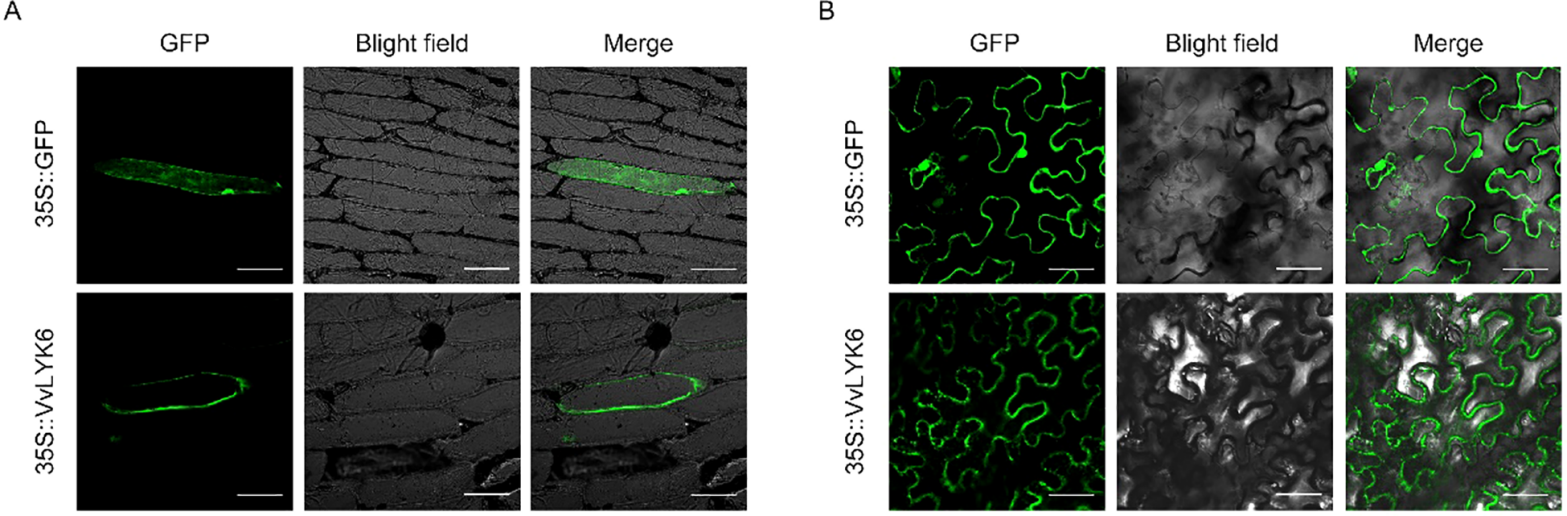
Subcellular localization of VvLYK6. **(A)** Subcellular localization of VvLYK6 in onion epidermal cells. The empty vector pRTL2 was used as a control. Scale bar = 100 μm. **(B)** Subcellular localization of VvLYK6 in *N. benthamiana* leaves. The empty vector pBGFP4 was used as a control. Scale bar = 50 μm. The transformed samples were observed using a laser scanning confocal microscope at 200 × magnification, and the excitation and emission wavelengths were 488 nm and 510 nm, respectively.

The coding sequence of *VvLYK6*, which spans 1869 bp and encodes 622 amino acids, was cloned using the cDNA from GF. A TMHMM-2.0 analysis predicted a transmembrane region between amino acids 256 and 278 (**Supplementary Figure 4**). VvLYK6 gene was cloned into the pRTL2 vector and introduced it into onion epidermal cells using a gene gun. After 24 h, confocal fluorescence microscopy confirmed that the PRTL2-*VvLYK6* fusion protein was localized exclusively to the plasma membrane, while the *GFP* control was distributed throughout the cell (**Figure 3A**). Additionally, the *VvLYK6* gene was inserted into the pBGFP4 plasmid to construct an expression vector, which was introduced into *A. tumefaciens* GV3101. This fusion vector was injected into *N. benthamiana* leaves, and after 24 h of incubation in the dark, fluorescence microscopy revealed that the pBGFP4-*VvLYK6* fusion protein was localized exclusively on the plasma membrane unlike the *GFP* control (**Figure 3B**). These results confirm that VvLYK6 is localized to the plasma membrane.

### 3.4 Functional validation of VvLYK6 in mediating white rot resistance

**Figure 4:**
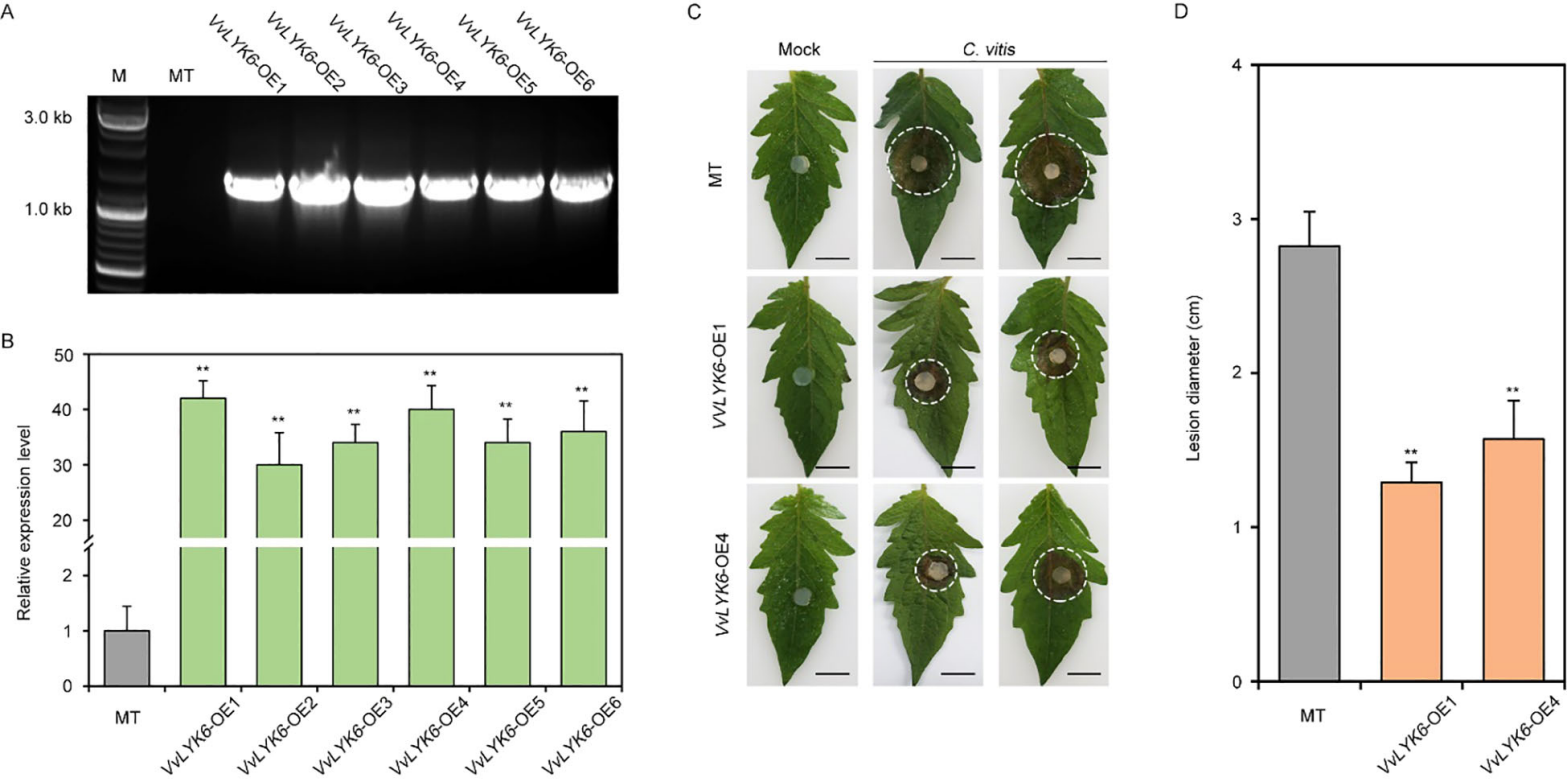
Identification of white rot resistance in tomato mediated by *VvLYK6*. **(A)** PCR products targeting a fragment of *VvLYK6* amplified from DNA extracted from *VvLYK6*-overexpression lines (*VvLYK6*-OE1-6) and tomato cv. Micro-Tom (MT). **(B)** Expression levels of *VvLYK6* gene in MT and *VvLYK6*-OE1–6 lines. **(C)** Resistance phenotypes of MT, *VvLYK6*-OE1, and -OE4 lines after 72 h inoculation with *C. vitis*. **(D)** Statistical analysis of lesion diameters in MT, *VvLYK6*-OE1, and -OE4 lines after 72 h inoculation with *C. vitis*. Three biological replicates were taken from each experiment, and six leaves were taken from each replicate. RT-qPCR was used to detect *VvLYK6* expression levels, which were calculated using 2^-ΔΔCT^ method with *S. lycopersicum GAPDH* as the internal reference gene. White dashed circles are used to mark the boundaries of diseased areas on tomato leaves. Scale bar = 1 cm. The error bar represents the standard error of three biological replicates, and the double asterisks represents a significant difference at the P < 0.01 level by t-test.

The calli system is an effective tool and has been successfully applied in studies on grapevine white rot resistance gene validation (Zhang et al., 2023b). Overexpression of *VvLYK6* in the grapevine cv. Thompson seedless calli resulted in a 45% reduction in the diameter of the colony compared to the control calli (**Supplementary Figure 5**). Concurrently, transient overexpression of *VvLYK6* in RG leaves resulted in a lesion diameter of 32% relative to controls upon inoculation with *C. vitis*, with significantly attenuated disease symptoms (**Supplementary Figure 6**). To further validate the role of *VvLYK6* in resistance to white rot, the gene was overexpressed in tomato cv. Micro-Tom, and overexpression lines were generated (**Figure 4A**). RT-qPCR showed the highest expression levels of *VvLYK6* in the *VvLYK6-*OE1 and *VvLYK6-*OE4 lines (**Figure 4B**). Western blot analysis using anti-FLAG antibodies confirmed stable overexpression of the VvLYK6-FLAG fusion protein in *VvLYK6*-OE1 and *VvLYK6*-OE4 lines, with no signal detected in the control (**Supplementary Figure 7**). Inoculation with *C. vitis* showed that the severity of disease was significantly reduced in the *VvLYK6-*OE1 and *VvLYK6-*OE4 lines compared to the MT plants, with lesion diameters reduced by 54% and 44%, respectively (**Figures 4C, D**). These results demonstrate that *VvLYK6* can improve the resistance to white rot.

### 3.5 SlLYK1 is involved in the resistance to white rot disease mediated by VvLYK6 in tomato

**Figure 5:**
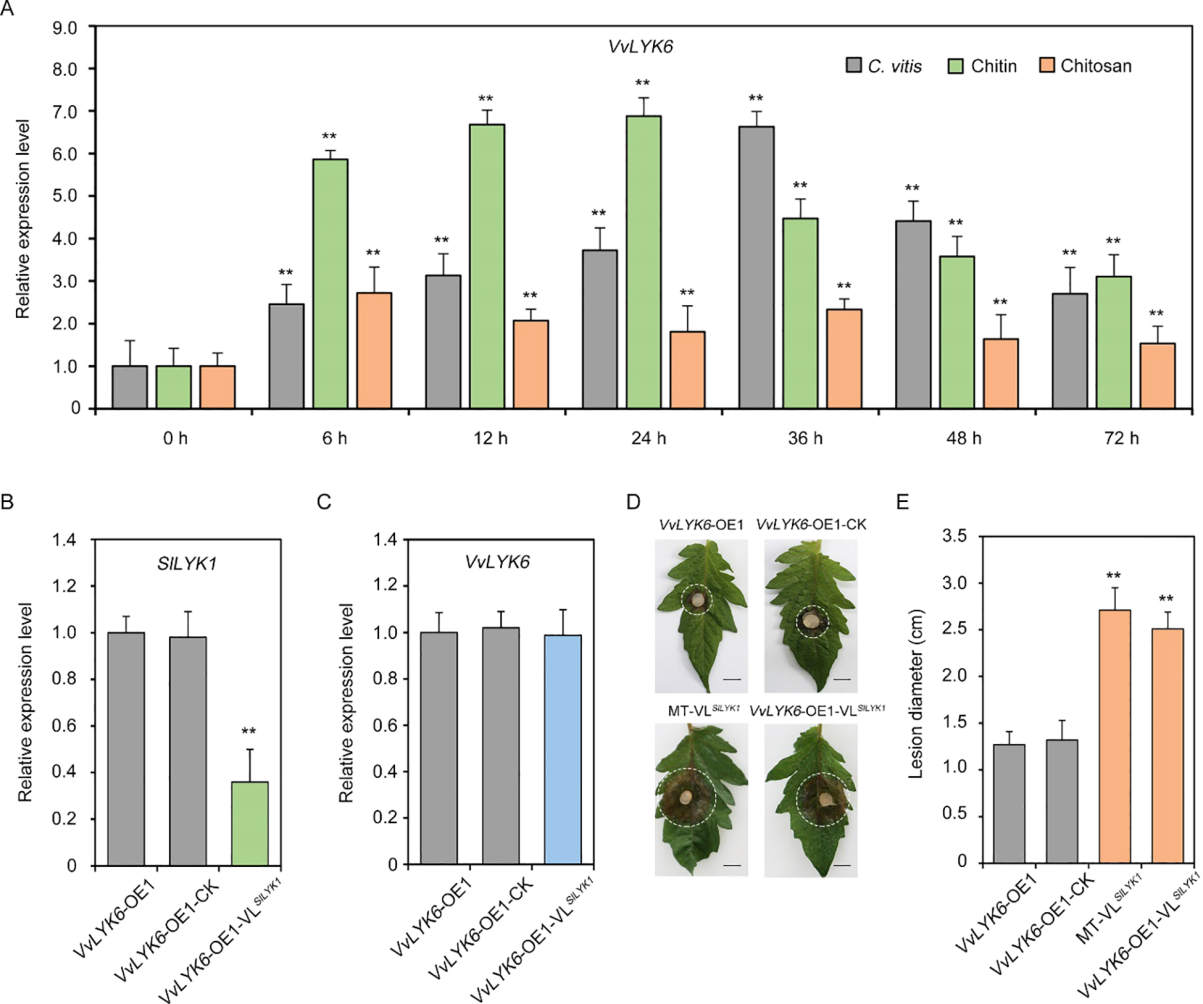
Tomato *SlLYK1* is involved in *VvLYK6*-mediated resistance to *C. vitis*. **(A)** The expression pattern of *VvLYK6* gene under the conditions of chitin (DP6), chitosan (DP6) and *C. vitis* treatment in GF. **(B)** SlLYK1 gene expression in *VvLYK6*-overexpression line (*VvLYK6*-OE1), *VvLYK6*-OE1 with the empty vector pTRV2:00 line (*VvLYK6*-OE1-pTRV2:00, *VvLYK6*-OE1-CK), and *VvLYK6*-OE1 with *SlLYK1*-silenced line (*VvLYK6*-OE1-pTRV2:*SlLYK1*, *VvLYK6*-OE1-VL*^SlLYK1^*). **(C)** VvLYK6 gene expression in *VvLYK6*-OE1, *VvLYK6*-OE1-CK, and *VvLYK6*-OE1-VL*^SlLYK1^* lines. **(D)** Resistance phenotypes of *VvLYK6*-OE1, *VvLYK6*-OE1-CK, *VvLYK6*-OE1-VL*^SlLYK1^* and tomato wild-type cv. Micro-Tom with *SlLYK1*-silenced line (MT-VL*^SlLYK1^*) lines after 72 h inoculation with *C. vitis*. **(E)** Statistical analysis of lesion diameter in *VvLYK6*-OE1, *VvLYK6*-OE1-CK, *VvLYK6*-OE1-VL*^SlLYK1^* and MT-VL*^SlLYK1^* lines after 72 h inoculation with *C. vitis*. Three biological replicates were taken from each experiment, and six leaves were taken from each replicate. RT-qPCR was used to detect *SlLYK1* and *VvLYK6* genes expression level, which were evaluated by 2^-ΔΔCT^ using *S. lycopersicum GAPDH* and *V. vinifera Actin-7* as the internal reference gene, respectively. White dashed circles are used to mark the boundaries of diseased areas on tomato leaves. Scale bar = 1 cm. The error bar represents the standard error of three biological replicates, and the double asterisks represents a significant difference at the P < 0.01 level of the t-test.

The LYK family genes are involved in the immune response induced by chitin and chitosan, a deacetylated derivative of chitin, and have been shown to be most effective in triggering immune response in grapevine when the polymer has a degree of polymerization six (DP6) (Brulé et al., 2019). In this study, the expression pattern of *VvLYK6* was investigated under treatment with chitin (DP6), chitosan (DP6) and *C. vitis*. The results showed that *C. vitis* infection gradually induced *VvLYK6* expression, which peaked at 36 hpi. Interestingly, chitin treatment also significantly upregulated *VvLYK6* expression, which progressively increased from 0 to 24 h and remained a high level. In contrast, chitosan elicited a markedly weaker induction of *VvLYK6* compared with both chitin and *C. vitis* inoculation. These results indicate that the *VvLYK6* gene is involved in chitin mediated immune responses (**Figure 5A**). As a co-receptor, *LYK1* plays a central role in mediating chitin-induced signal transduction with the LYK family, and *SlLYK1* is also necessary for the chitin induced fungal resistance in tomato (Jaiswal et al., 2022). To investigate the role of *LYK1* in *LYK6*-mediated white rot resistance, we used VIGS to silence *SlLYK1* in tomato, and the silencing efficiency reached 65% (**Figure 5B**). The expression pattern of *VvLYK6* in the *VvLYK6-*OE1, *VvLYK6-*OE1-CK, and *VvLYK6-*OE1-VL*^SlLYK1^* lines showed no significant difference following inoculation with *C. vitis*, indicating that silencing *SlLYK1* did not affect the expression of *VvLYK6* (**Figure 5C**). Following inoculation with *C. vitis* for 72 h, the MT-VL*^SlLYK1^* and *VvLYK6-*OE1-VL*^SlLYK1^* lines had a lesion diameter of 2.71 cm and 2.51 cm, which was significantly larger than the 1.27 cm and 1.32 cm observed in the *VvLYK6-*OE1 and *VvLYK6-*OE1-CK lines, respectively (**Figures 5D, E**). These results suggest that *SlLYK1* is involved in the resistance to white rot disease in tomato that is mediated by *VvLYK6*.

### 3.6 Activation of the SA signaling pathway by VvLYK6 in response to white rot

**Figure 6:**
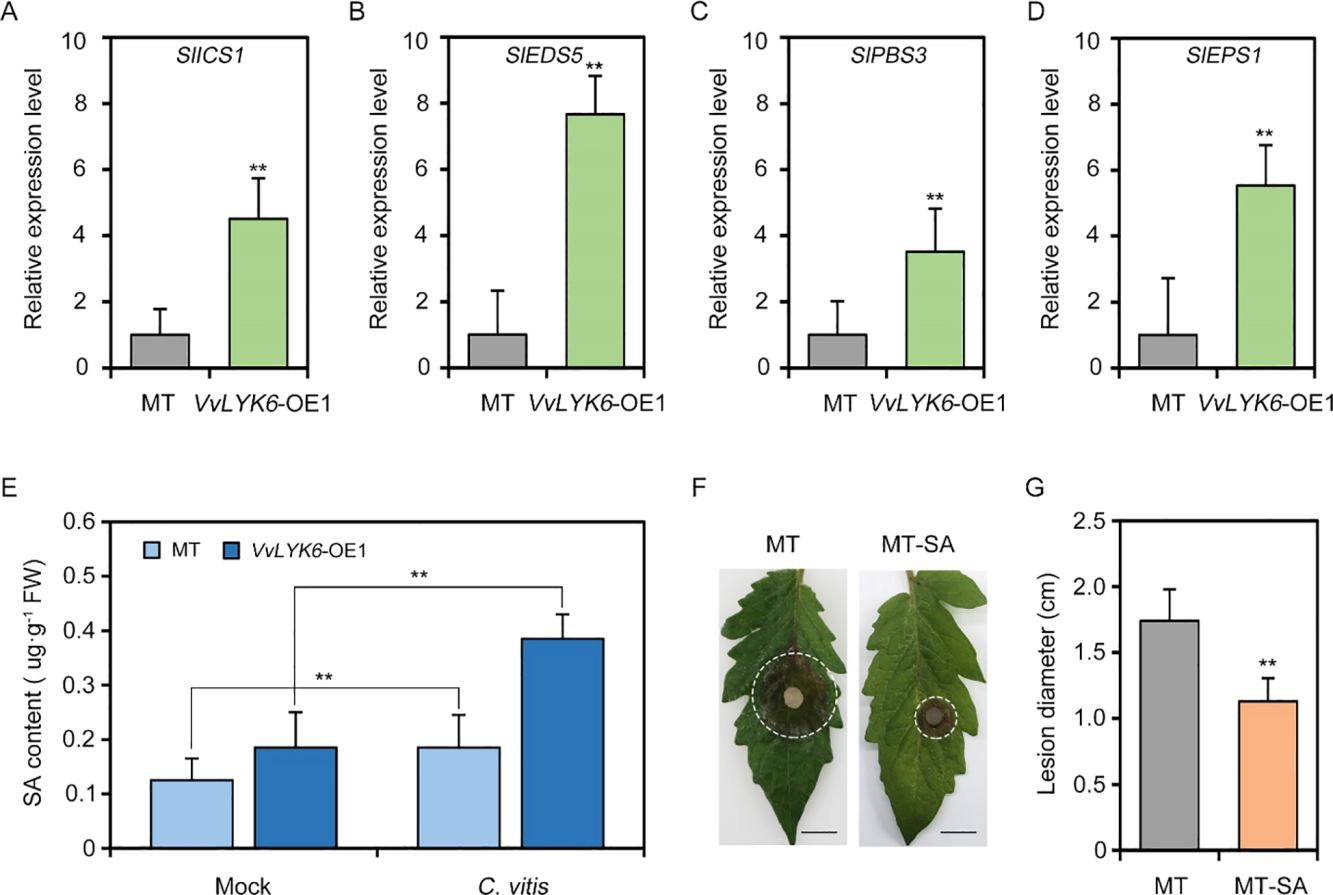
VvLYK6 mediates resistance to white rot through the salicylic acid (SA) signaling pathway. Expression patterns of isochorismate synthase (ICS) pathway of SA biosynthesis genes, including *SlICS1* **(A)**, *SlEDS5* **(B)**, *SlPBS3* **(C)**, and *SlEPS1* **(D)** in tomato wild-type cv. Micro-Tom (MT) and *VvLYK6*-overexpression line (*VvLYK6*-OE1) after 24 h inoculation with *C. vitis*. **(E)** SA content in MT and *VvLYK6*-OE1 line after 24 h inoculation with *C. vitis*. **(F)** Resistance phenotypes of MT and MT treated with SA (MT-SA) leaves after 72 h inoculation with *C. vitis*. **(G)** Statistical analysis of lesion diameter in MT and MT-SA leaves after 72 h inoculation with *C. vitis*. Three biological replicates were taken from each experiment, and six leaves were taken from each replicate. RT-qPCR was used to detect the expression level of candidate genes, which was evaluated using the 2^-ΔΔCT^ method with *S. lycopersicum GAPDH* as the internal reference gene. White dashed circles are used to highlight the boundaries of diseased areas on tomato leaves. Scale bar = 1 cm. Error bars represent the standard error of three biological replicates, and the double asterisks represent a significant difference at the P < 0.01 level in the t-test.

It is known that *ICS1, EDS5, PBS3*, and *EPS1* are the key genes in ICS pathway, which is responsible for 90% of the biosynthesis of SA (**Supplementary Figure 8**) (Garcion et al., 2008). In this study, these genes were significantly upregulated in the *VvLYK6*-OE1 line following inoculation with *C. vitis* (**Figures 6A–D**). The SA signaling pathways is critical for the immune response of plants. After 24 h of *C. vitis* inoculation, the levels of SA in the *VvLYK6*-overexpression tomato line (*VvLYK6*-OE1) was significantly higher compared to the tomato wild-type cv. Micro-Tom (MT) plants (**Figure 6E**). Additionally, the MT plants that were treated with exogenous SA and then inoculated with *C. vitis* displayed reduced lesion sizes and improved resistance after 72 h (**Figures 6F, G**). These results indicate that *VvLYK6* enhances resistance to white rot by activating both SA synthesis and SA signaling genes in tomato.

### 3.7 VvLYK6-mediated immune response in tomato

**Figure 7:**
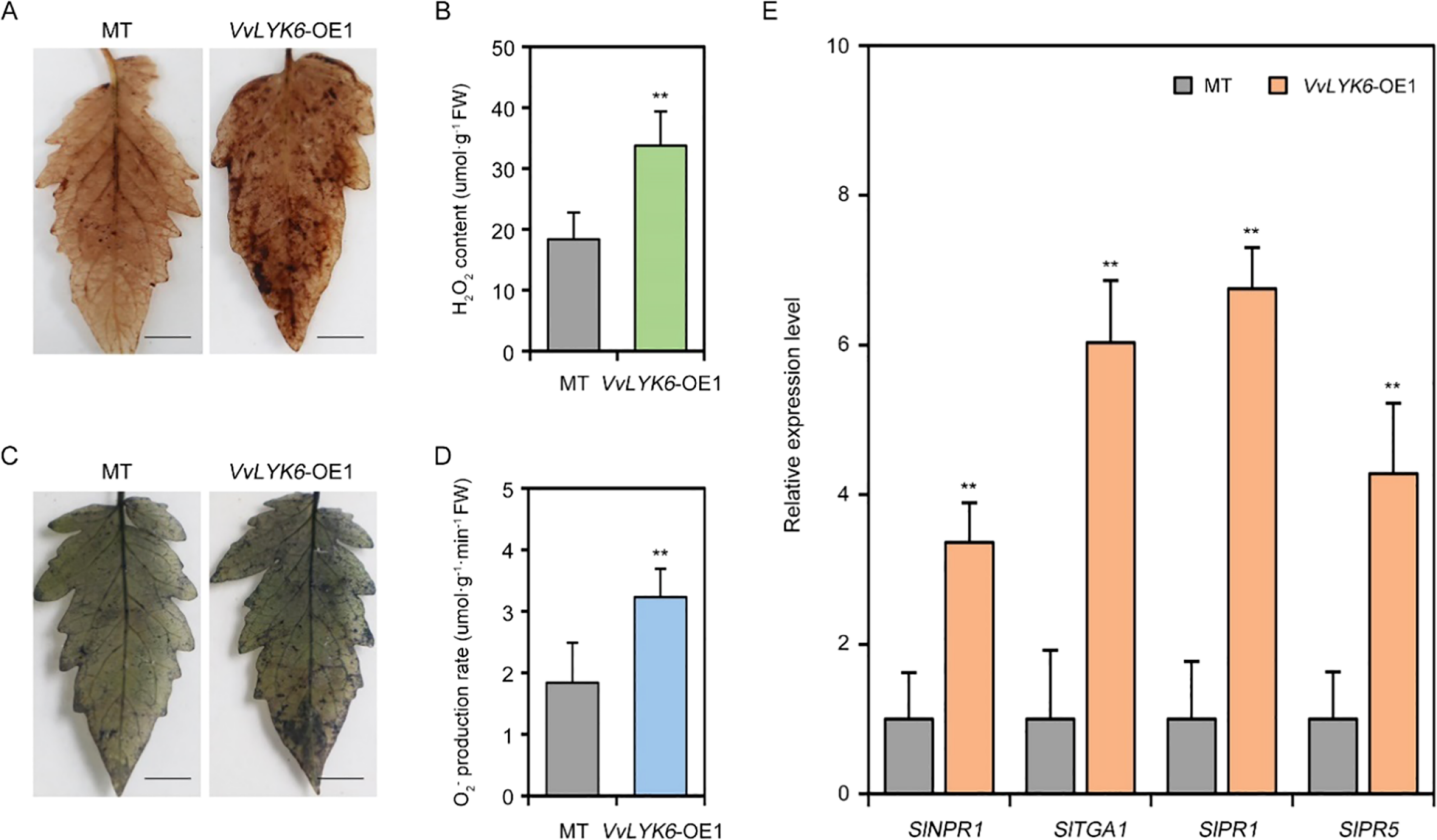
VvLYK6 mediates resistance to white rot by inducing a reactive oxygen species (ROS) burst and activating the expression of pathogenesis-related (PR) genes. **(A)** H_2_O_2_ accumulation in tomato wild-type cv. Micro-Tom (MT) and *VvLYK6*-overexpression line (*VvLYK6*-OE1) after 24 h inoculation with *C. vitis*. Three biological replicates were taken from each experiment, and four leaves were taken from each replicate. **(B)** Content of H_2_O_2_ in MT and *VvLYK6*-OE1 after 24 h inoculation with *C. vitis*. **(C)** H_2_O_2_ accumulation of MT and *VvLYK6*-OE1 after 24 h inoculation with *C. vitis*. Three biological replicates were taken from each experiment, and four leaves were taken from each replicate. **(D)** Superoxide anion (O_2_^-^) content in MT and *VvLYK6*-OE1 after 24 h inoculation with *C. vitis*. **(E)** Expression patterns of PR genes in the SA signaling pathway in MT and *VvLYK6*-OE1 after 24 h inoculation with *C. vitis*. RT-qPCR was used to detect candidate gene expression levels, which was evaluated using the 2^-ΔΔCT^ method with *S. lycopersicum GAPDH* as the internal reference gene. Scale bar = 1 cm. Error bars represent the standard error of three biological replicates, and the double asterisks represent a significant difference at the P < 0.01 level by t-test.

To investigate how *VvLYK6* mediates resistance to white rot, the ROS burst was analyzed 12 h after inoculation with *C. vitis* in both the MT plants and *VvLYK6*-OE1 line. The leaves in the *VvLYK6*-OE1 line browned significantly more and had levels of hydrogen peroxide that were 1.84-folds higher than those in MT plants (**Figures 7A, B**). Additionally, the leaves in the *VvLYK6*-OE1 line stained blue more intensely, and there was 1.76-fold higher production rate of production of superoxide anions in *VvLYK6*-OE1 line than in the MT plants (**Figures 7C, D**). The analysis of the expression levels of the PR genes related to the SA signaling pathway after 12 h of inoculation with *C. vitis* revealed that *SlNPR1, SlTGA1, SlPR1*, and *SlPR5* genes were all upregulated in the *VvLYK6*-OE1 line compared to MT plants, and *SlPR1* and *SlPR5* increased significantly by 6.75- and 4.28-fold, respectively (**Figure 7E**). These findings suggest that *VvLYK6* enhances resistance to white rot by inducing the ROS burst and upregulating expression of the PR genes in tomato.

## 4 Discussion

Plants often encounter pathogens during their growth, and infection can lead to disease; thus, plants have evolved immune systems to resist attack by pathogens (Li et al., 2021a). Typically, upon pathogen contact with the cell surface, the host perceives PAMPs via membrane-anchored receptor proteins, which initiate PTI (Yuan et al., 2021). When pathogens secrete effectors into the cell, the host activates resistance (R) genes, which trigger ETI (Yuan et al., 2021). The LYK family is an essential group of membrane receptor proteins in plants (Chen et al., 2020b). In grapevine, the VvLYK family includes 15 genes (Brulé et al., 2019). Transcriptome sequencing revealed that *VvLYK6* is significantly induced in GF upon inoculation with *C. vitis*, which suggests that it is involved in grapevine resistance to white rot (**Figure 1**). The silencing of *VvLYK6* in GF increased susceptibility to white rot, while its overexpression in grapevine cv. Thompson seedless calli, RG leaves, and tomato cv. Micro-Tom enhanced resistance, demonstrating that *VvLYK6* functions in mediating resistance to white rot (**Figures 2**, **3**). VvLYK6 is the first gene in the VvLYK family identified to have a role in white rot resistance, and it provides a new target for the genetic improvement of disease resistance.

This study showed that VvLYK6 is localized to the plasma membrane, which is consistent with the known function of the LYK family in recognizing chitin and its derivatives to activate immune signaling pathways and mediate resistance responses (**Figure 4**). The expression of *VvLYK6* was significantly induced by treatment with chitin, which is consistent with its expression pattern following inoculation with *C. vitis.* This suggests that *VvLYK6* may mediate the immune responses of grapevine to *C. vitis* by recognizing chitin (**Figure 5**). Additionally, chitin can induce the interaction between VvLYK5–1 and VvLYK1-1, which suggests that VvLYK1–1 may be a co-receptor in the LYK family of grapevine and mediate PAMP perception (Brulé et al., 2019). This would be similar to the role of CERK1 in *A. thaliana* and OsCERK1 in rice (*O. sativa*) (Erwig et al., 2017; Wang et al., 2024). To verify whether the *VvLYK6*-mediated immune responses require a co-receptor, this study silenced the *SlLYK1* gene in the tomato *VvLYK6*-overexpression line (*VvLYK6*-OE1). Although the expression levels of *VvLYK6* were not significantly different between the *VvLYK6*-OE1 and *SlLYK1*-silenced lines, the silenced lines were significantly more susceptible to *C. vitis*. The results indicate that *SlLYK1* is required for *VvLYK6*-mediated white rot resistance in tomato (**Figure 5**). However, further validation is needed to confirm whether SlLYK1 directly interacts with VvLYK6 to form a module that regulates immune responses to *C. vitis*.

The LYK family has been widely recognized as receptor kinases with pathogen recognition functions in plants, which induce stress responses, such as a ROS burst, and activate intracellular immune signaling pathways, thereby initiating downstream defense responses upon PAMP recognition (Richards and Rose, 2019). In plants, the JA and SA pathways are the main immune signaling pathways and mediate resistance to necrotrophic and biotrophic/semi-biotrophic pathogens, respectively (Vlot et al., 2009). C. vitis is a semi-biotrophic pathogen (Li et al., 2023b; Rahman et al., 2022). This study found that the overexpression of *VvLYK6* in tomato significantly activated the expression of genes related to SA biosynthesis, including *SlICS1, SlEDS5, SlPBS3*, and *SlEPS1* (**Figure 6**). Additionally, SA content was significantly increased in tomato lines that overexpressed *VvLYK6*, and exogenous SA treatment of tomato leaves significantly enhanced resistance to *C. vitis* (**Figure 6**). These findings indicate that *VvLYK6* mediates resistance to white rot by activating the SA signaling pathway in tomato. Li et al. (2023b) found that grapevine *VvTGA8* participates in the activation of the SA signaling pathway and mediates resistance to white rot by inducing the expression of related PR genes, which is consistent with the conclusions of this study. To our knowledge, this is the first study to demonstrate that a grapevine VvLYK family gene (*VvLYK6*) mediates resistance via the SA signaling pathway.

The ROS burst is a major stress response in plants, and it is widely present in almost all higher plants (Cao et al., 2014). Reports have shown that unlike *A. thaliana* and rice, chitosan cannot induce the production of hydrogen peroxide in grapevine (Trdá et al., 2014). However, this study found that the tomato overexpression line harboring *VvLYK6* significantly induced the accumulation of hydrogen peroxide and the generation of superoxide anions after inoculation with *C. vitis* (**Figure 7**). This suggests that *VvLYK6* may activate the production of hydrogen peroxide by recognizing other PAMPs from *C. vitis*. After downstream effector proteins decode the immune signals, defense responses, including the expression of defense genes, are activated, thereby mediating resistance in plants (Roudaire et al., 2023). AtLYK2 and *AtLYK4* in *A. thaliana* can mediate resistance to *B. cinerea* and *P. syringae* by inducing the expression of PR genes, while AtLYK3 negatively regulates PR gene expression to suppress resistance to *B. cinerea* and *P. carotovorum* (Paparella et al., 2014). VvLYK1–1 and *VvLYK5–1* in grapevine have been shown to mediate resistance in *A. thaliana* mutants by inducing the expression of PR genes (Brulé et al., 2019; Roudaire et al., 2023). In this study, *VvLYK6* was found to mediate immune signals through the SA signaling pathway and further activate the expression of PR genes related to the SA pathway in tomato, including *SlPR1* and *SlPR5* (**Figure 7**), which is consistent with the immune responses reported in other species, such as rice (*O. sativa*), apple (*Malus* × *domestica*), and strawberry (*Fragaria* × *ananassa*) (Chen et al., 2020a; Kaku et al., 2006; Zhang et al., 2023a). However, its role in other plant-pathogen interactions is less clear. For example, Villette et al. (2025) showed that *VvLYK6* can attenuate chitin-triggered immune responses, and that its constitutive expression in *Arabidopsis* increased susceptibility to necrotrophic fungi such as *B. cinerea* and *Alternaria brassicicola*, while having limited impact on oomycete infection. These observations point to a context-dependent function of *VvLYK6* that may differentially influence immune outputs depending on pathogen lifestyle and signaling background.

## 5 Conclusions

In summary, this study identifies *VvLYK6* as a key gene that mediates resistance to white rot. VvLYK6 responds to *C. vitis* inoculation by activating the SA signaling pathway and upregulating the PR genes related to the SA pathway, as well as inducing an ROS burst, which collectively enhance resistance to white rot. This gene represents a promising target for improving grapevine resistance, although its use in breeding will require validation of functional allelic variation across diverse germplasm. Its role in salicylic acid signaling and ROS accumulation also suggests potential growth-defense trade-offs that merit further study.

## Data Availability

The data presented in the study are deposited in the NCBI Sequence Read Archive (SRA) repository, accession numbers PRJNA995417 and PRJNA1001063.
