# Supplementary material for: Grapevine LysM-containing receptor-like kinase VvLYK6 enhances resistance to white rot in tomato
Source: Front Plant Sci. 2026 Jun 9;17:1869419. doi: 10.3389/fpls.2026.1869419 (PMC13286899; doi:10.3389/fpls.2026.1869419)
Supplement: Supplementary file 1 [file DataSheet1.zip › Supplementary files_R1/Supplementary Figure 1-8.docx]

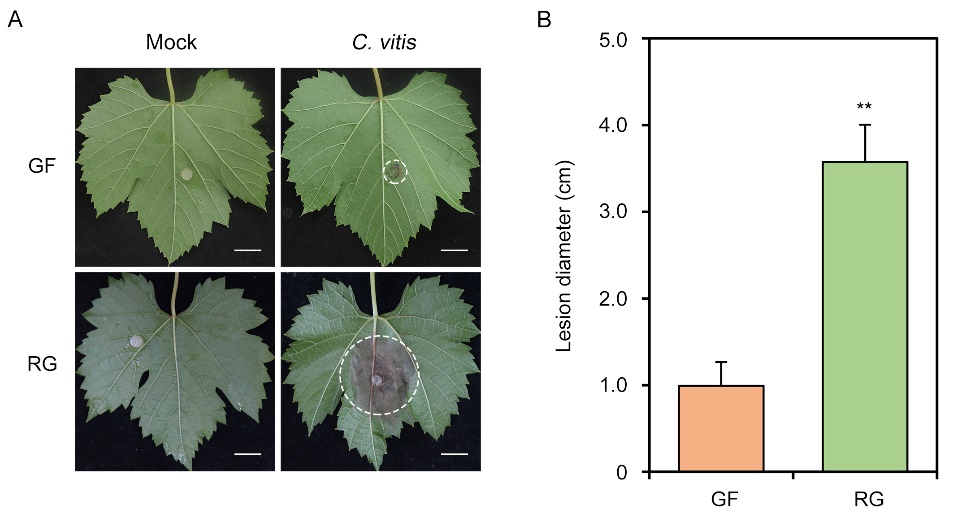


**Supplementary FIGURE 1 Resistance phenotype of grapevine response to *C. vitis*.** (A) Resistance identification of grapevine cv. Guifeimeigui (GF) and cv. Red Globe (RG) to *C. vitis*. (B) Statistical analysis of lesion diameter in GF, and RG after 72 h inoculation with *C. vitis*. White dashed circles are used to mark the boundaries of diseased areas on grapevine leaves. Scale bar = 1 cm. The error bar represents the standard error of three biological replicates, and the double asterisks represents a significant difference at the P < 0.01 level of the t-test.


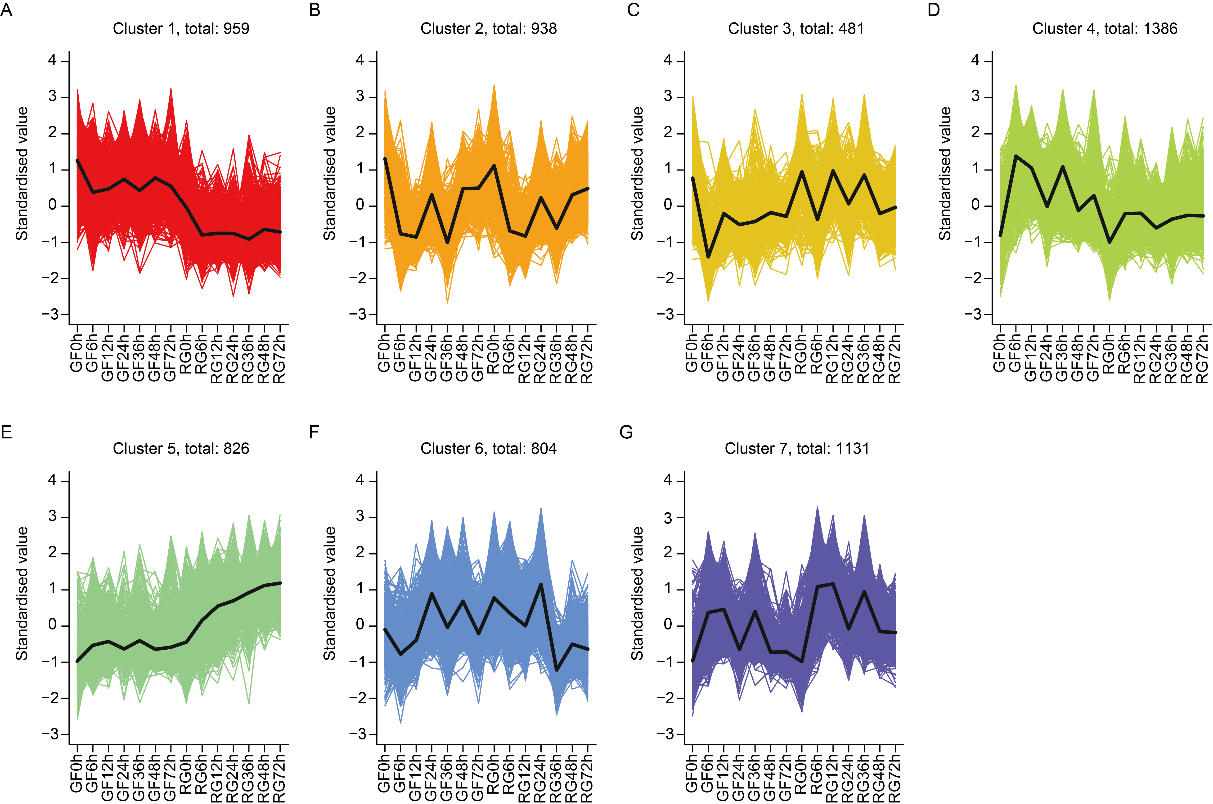


**Supplementary FIGURE 2 Dynamics of gene expression after inoculation with *C. vitis*.** K-means clustering grouped the differentially expressed genes (DEGs) of the grapevine GF and RG into seven clusters. The x-axis depicts at 6, 12, 24, 36, 48 and 72 h time-points after *C. vitis* inoculation, and the y-axis depicts the Z-score standardized per gene.


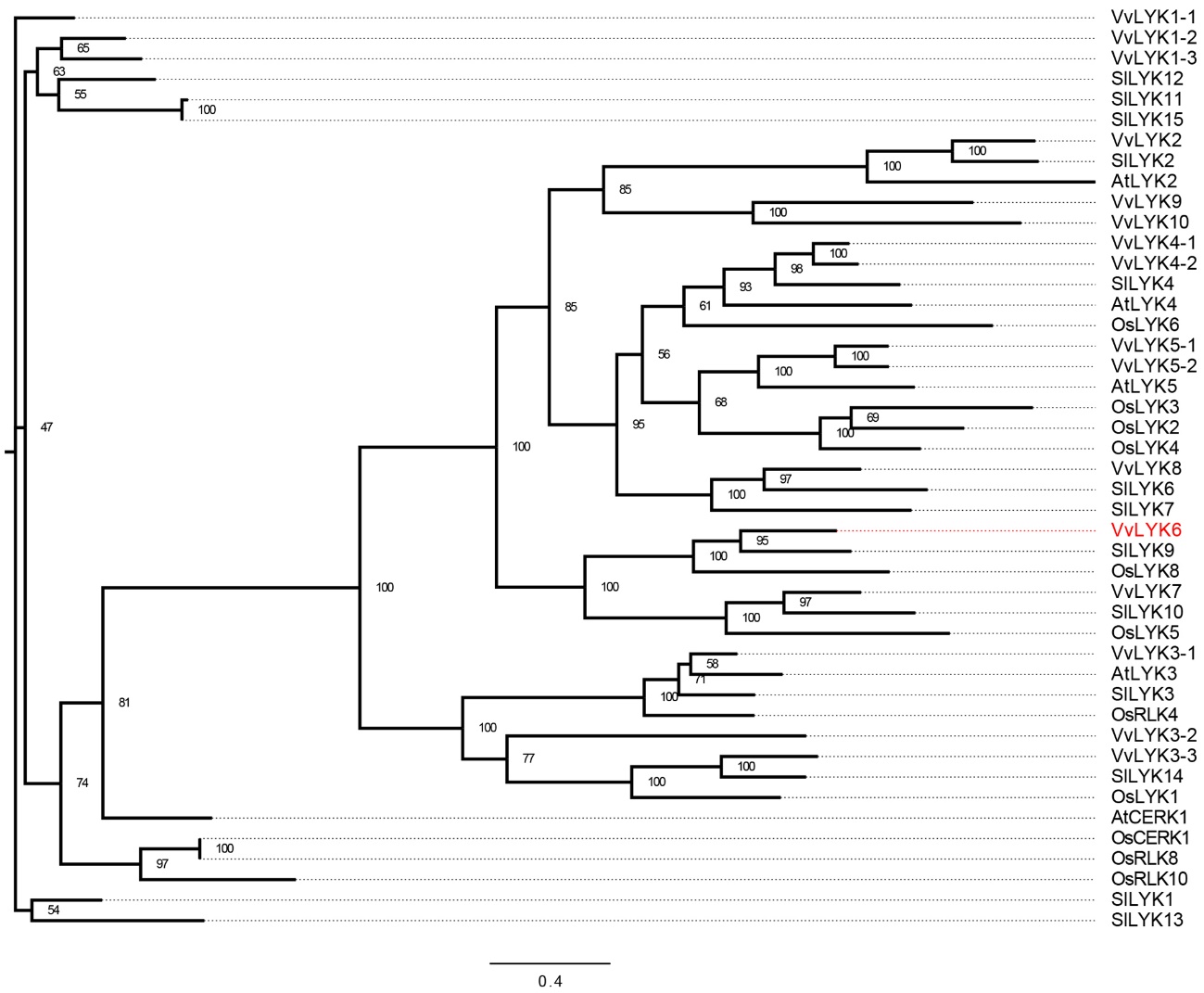


**Supplementary FIGURE 3 Phylogenetic analysis of LYKs in different species.** The 1,000-bootstrap maximum-likelihood phylogenetic tree was constructed with software IQ-TREE. LYKs from *V. vinifera*, *A. thaliana*, *S. lycopersicum*, and *O. sativa* are listed in Supplementary Table 8. VvLYK6 is highlighted in red.


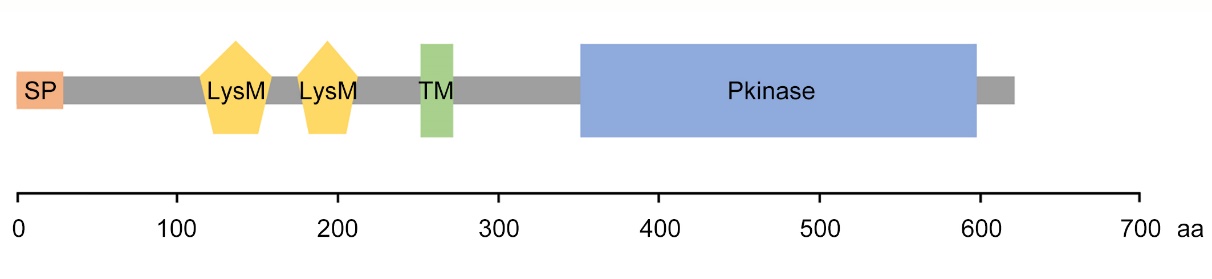


**Supplementary FIGURE 4 Domain composition model of VvLYK6.** The size and location of the domains contained in VvLYK6 gene, including the signal peptide (SP), LysM domains (LysM), transmembrane domain (TM), and kinase domain (Pkinase), were labeled in the model.


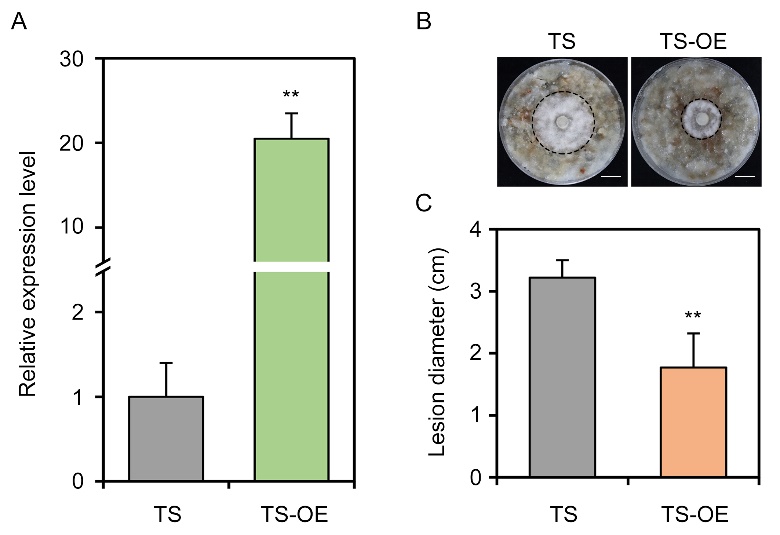


**Supplementary FIGURE 5 Identification of white rot resistance in grapevine** **calli mediated by *VvLYK6*.** (A) VvLYK6 gene expression pattern in grapevine Thompson seedless calli (TS) and *VvLYK6*-overexpression calli (TS-OE). (B) Resistance phenotypes of TS, and TS-OE after 72 h inoculation with *C. vitis*. (C) Statistical analysis of lesion diameter in TS, and TS-OE after 72 h inoculation with *C. vitis*. RT-qPCR was used to detect *VvLYK6* gene expression levels, which was evaluated using the 2^-ΔΔCT^ method with *V. vinifera Actin-7* as the internal reference gene. Black dashed circles are used to highlight the boundaries of diseased areas on grapevine leaves. Scale bar = 1 cm. The error bars represent the standard error of three biological replicates, and the double asterisks represent a significant difference at the P < 0.01 level in the T-test.


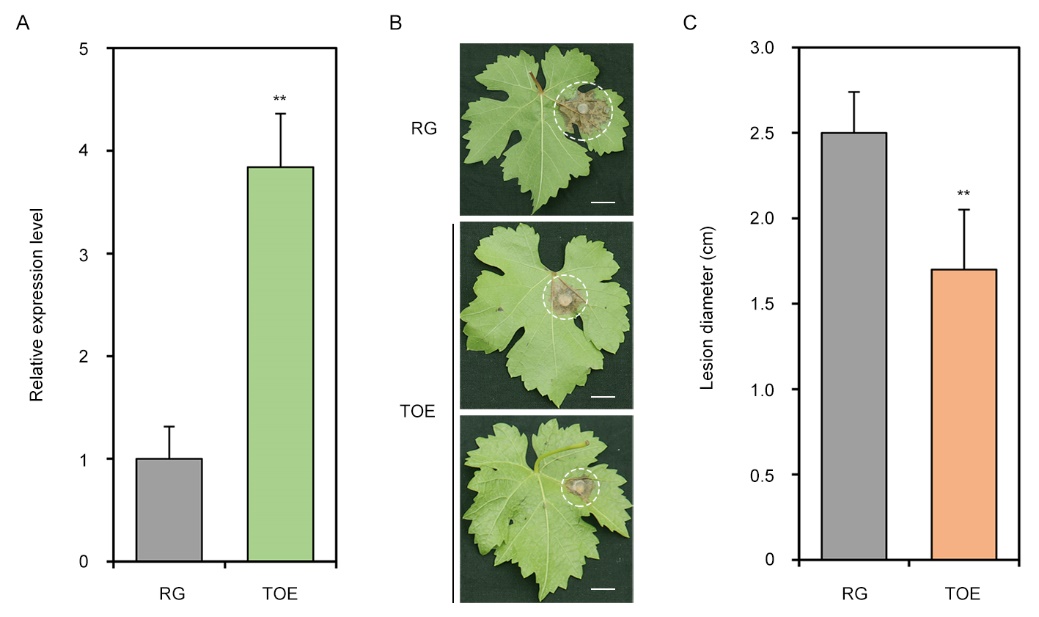


**Supplementary FIGURE 6 Identification of white rot resistance in grapevine** **leaves mediated by *VvLYK6*.** (A) VvLYK6 gene expression pattern in grapevine RG and *VvLYK6* transient overexpression leaves (TOE). (B) Phenotypes of RG, and TOE after 72 h inoculation with *C. vitis*. (C) Statistical analysis of lesion diameter in RG, and TOE after 72 h inoculation with *C. vitis*. RT-qPCR was used to detect *VvLYK6* gene expression levels, which was evaluated using the 2^-ΔΔCT^ method with *V. vinifera Actin-7* as the internal reference gene. White dashed circles are used to highlight the boundaries of diseased areas on grapevine leaves. Scale bar = 1 cm. The error bars represent the standard error of three biological replicates, and the double asterisks represent a significant difference at the P < 0.01 level in the t-test.


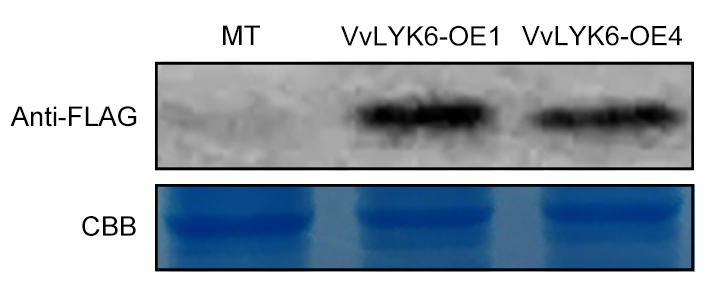


**Supplementary FIGURE 7 Detection of VvLYK6-FLAG protein in tomato cv. Micro-Tom (MT) and *VvLYK6*-overexpression lines (*VvLYK6*-OE1/4).** Protein samples were separated on SDS-PAGE, transferred to PVDF membranes using wet transfer. Membranes were blocked with 5% skim milk, incubated with primary antibodies, followed by HRP-conjugated secondary antibodies. Protein bands were visualized using ECL kit (Thermo Fisher, USA) and imaged on Tanon 5200 (Shanghai, China).


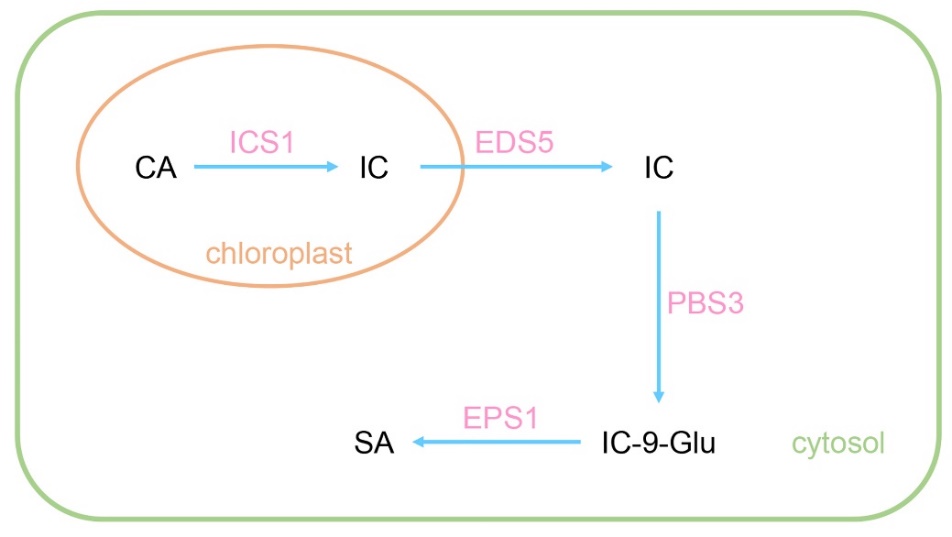


**Supplementary FIGURE 8 Isochorismate synthase (ICS) pathway of SA biosynthesis.** ICS1, *EDS5*, *PBS3*, and *EPS1* are key genes in ICS pathway of SA biosynthesis.
